# Alpha-to-beta- and gamma-band activity reflect predictive coding in affective visual processing

**DOI:** 10.1038/s41598-021-02939-z

**Published:** 2021-12-06

**Authors:** Andreas Strube, Michael Rose, Sepideh Fazeli, Christian Büchel

**Affiliations:** Department of Systems Neuroscience, University Medical Center Hamburg-Eppendorf, 20246 Hamburg, Germany

**Keywords:** Neuroscience, Cognitive neuroscience, Emotion, Sensory processing, Visual system

## Abstract

Processing of negative affective pictures typically leads to desynchronization of alpha-to-beta frequencies (ERD) and synchronization of gamma frequencies (ERS). Given that in predictive coding higher frequencies have been associated with prediction errors, while lower frequencies have been linked to expectations, we tested the hypothesis that alpha-to-beta ERD and gamma ERS induced by aversive pictures are associated with expectations and prediction errors, respectively. We recorded EEG while volunteers were involved in a probabilistically cued affective picture task using three different negative valences to produce expectations and prediction errors. Our data show that alpha-to-beta band activity after stimulus presentation was related to the expected valence of the stimulus as predicted by a cue. The absolute mismatch of the expected and actual valence, which denotes an absolute prediction error was related to increases in alpha, beta and gamma band activity. This demonstrates that top-down predictions and bottom-up prediction errors are represented in typical spectral patterns associated with affective picture processing. This study provides direct experimental evidence that negative affective picture processing can be described by neuronal predictive coding computations.

## Introduction

People see hundreds of unfamiliar faces in daily life, while seeing famous faces is very rare and surprising and—in terms of predictive coding—unexpected, leading to a large prediction error. Utilizing time–frequency analysis of brain data, it has been shown for example that famous faces elicit larger gamma responses as compared to unfamiliar faces^1,2^.

Predictive coding of perception assumes that neuronal circuits implement perception and learning by constantly matching incoming sensory data with the top-down predictions of an internal or generative model^3–5^. Consequently, a system can refine models with better predictions by minimizing prediction errors regarding the sensory environment, leading to a more efficient encoding of information^6^.

The Free Energy principle including aspects of predictive coding specifically posits the minimization of “free energy” (and thus, indirectly prediction errors) as a mechanism to ensure that agents spend most of their time in a small number of valuable (i.e. positive) and expected states^6^. With regards to affective stimuli, this agrees with findings showing that visual stimuli with a negative valence (i.e. a negative and thus unexpected state) produce larger gamma responses than neutral and positive visual stimuli^7–16^. Results interpreting the effects of negative valence in the gamma band could be associated with the surprise (i.e. general low probability of a negative encounter) that negative stimuli entail. However, in most studies this cannot be disentangled from the valence as the prediction error associated with a negative stimulus per se cannot be disentangled from the prediction error in the individual experimental setting. To achieve this, additional prediction errors have to be introduced experimentally.

Within the framework of predictive coding, lower frequency oscillatory alpha-to-beta band activity has been linked to top-down predictive signals and higher frequency gamma band activity to bottom-up prediction errors^4,17,18^. Comparably, cortical dynamics induced by emotional picture processing comprise event-related desynchronization (ERD) in the alpha-to-beta band^19–28^ and event-related synchronization (ERS) in the gamma band^9,12,14,22,26,29–33^.

Alpha ERD (a decrease in power in the ~ 8–12 Hz range) following affective images is smaller when the image is anticipated, and the tendency is more prominent for images bearing negative emotional valence^34^. This might be interpreted as differences in the encoding of expectation signals in a predictive coding framework, where expectation signals manifest as increases in low frequency (alpha-to-beta) activity. In this context, predictive coding suggests that feed-forward prediction errors reflect the difference between top-down expectation signals (e.g. pre-activated neuronal units based on the anticipation of threat) and actual stimulus input^17^.

In the context of affective picture processing, it is interesting to note that participants with dysphoria elicit a smaller alpha ERD in response to pleasant pictures, but not to unpleasant pictures^24^. In the context of predictive coding, affective disorders (such as major depression) have been linked with bottom-up deficits in predictive processing and increased precision of negative prior beliefs^35^. The depressed phenotype may emerge from a collection of depressive beliefs associated with the causal structure of the world^36^. As a consequence, treating depression could be conceptualized as equipping the brain with the resources to modify its internal model of the world^37^. Hence, treatment of depression would be associated with brain's relevant statistical structures becoming “less pessimistic”^36^. Thus, the predictive coding model of emotional states associated with affective disorders might be of particular interest for mechanistic insights in depression, as it represents such an internal model with potentially pathological variations in its statistical structure^35–39^.

In summary, we hypothesize that alpha-to-beta ERD and gamma ERS typically found in responses to negative affective stimuli are actually signals related to predictive coding. This posits that alpha-to-beta ERD responses should be modulated by expectations, whereas gamma ERS responses should be modulated by prediction errors or surprise.

Consequently, we conducted a cue-stimulus paradigm to unravel predictive coding dynamics in affective picture processing. We specifically introduced prediction errors experimentally by presenting stimuli in two different modalities, pain and vision (i.e. affective pictures). In the affective picture part presented here, we presented emotionally negative stimuli and manipulated the degree of negative valence. Participants were asked to rate the valence of the content on a four-point rating scale. We expected the anticipated degree of the aversive content to be related to alpha-to-beta ERD. If surprise is a main driving factor of gamma ERS (as derived from a predictive coding perspective), we expected an increase of gamma power when there was a mismatch between the anticipated degree of aversion and the actual aversive quality of the picture. If the negative valence or aversive quality is contributing to the gamma ERS effect, we expected an increase of gamma power with higher aversion regardless of the anticipated degree of aversion. Finally, based on hypotheses regarding a negative valence associated with prediction errors^40^, we expected that a greater mismatch between predicted and actual valence elicits larger valence ratings.

## Methods

### Participants

We investigated 35 healthy male participants (mean 26, range 18–37 years), who were paid as compensation for their participation. Applicants were excluded if one of the following exclusion criteria applied: neurological, psychiatric, dermatological diseases, pain conditions, current medication, or substance abuse. All volunteers gave their informed consent. The study was approved by the Ethics board of the Hamburg Medical Association. Data from six participants had to be excluded from the final EEG data analysis due to technical issues during the EEG recording (i.e. the data of the excluded participants were contaminated with excessive muscle and/or technical artifacts) leaving a final sample of 29 participants.

### Stimuli and task

Stimulus properties were chosen to be identical to a previous fMRI study of predictive coding where both expectation and absolute prediction error effects were observed in pain^41^.

Aversive pictures were chosen from the International Affective Picture System (IAPS)^42^ database at three different levels of valence. The images presented during the EEG experiment had three levels of valence of which the low valence category had valence values of 2.02 ± 0.05 (mean ± standard error), the medium valence category had valence values of 4.06 ± 0.02 (mean ± standard error) and the high valence category had valence values of 5.23 ± 0.01 (mean ± standard error). The pain part of this data will not be described here, but has been reported in Strube et al., 2021^43^.

Prior to each picture or heat stimulus, a visual cue was presented. The color of the cue (triangle, visual angle of each side: 0.96°) indicated (probabilistically) the modality of the stimulus (orange for picture and blue for heat). A white digit depicted inside of each triangle indicated (probabilistically) the intensity of the subsequent stimulus (1, 2 and 3 for low, medium and high valence). During the whole trial, a centered fixation cross (visual angle: 0.24°) was presented on the screen.

**Figure 1:**
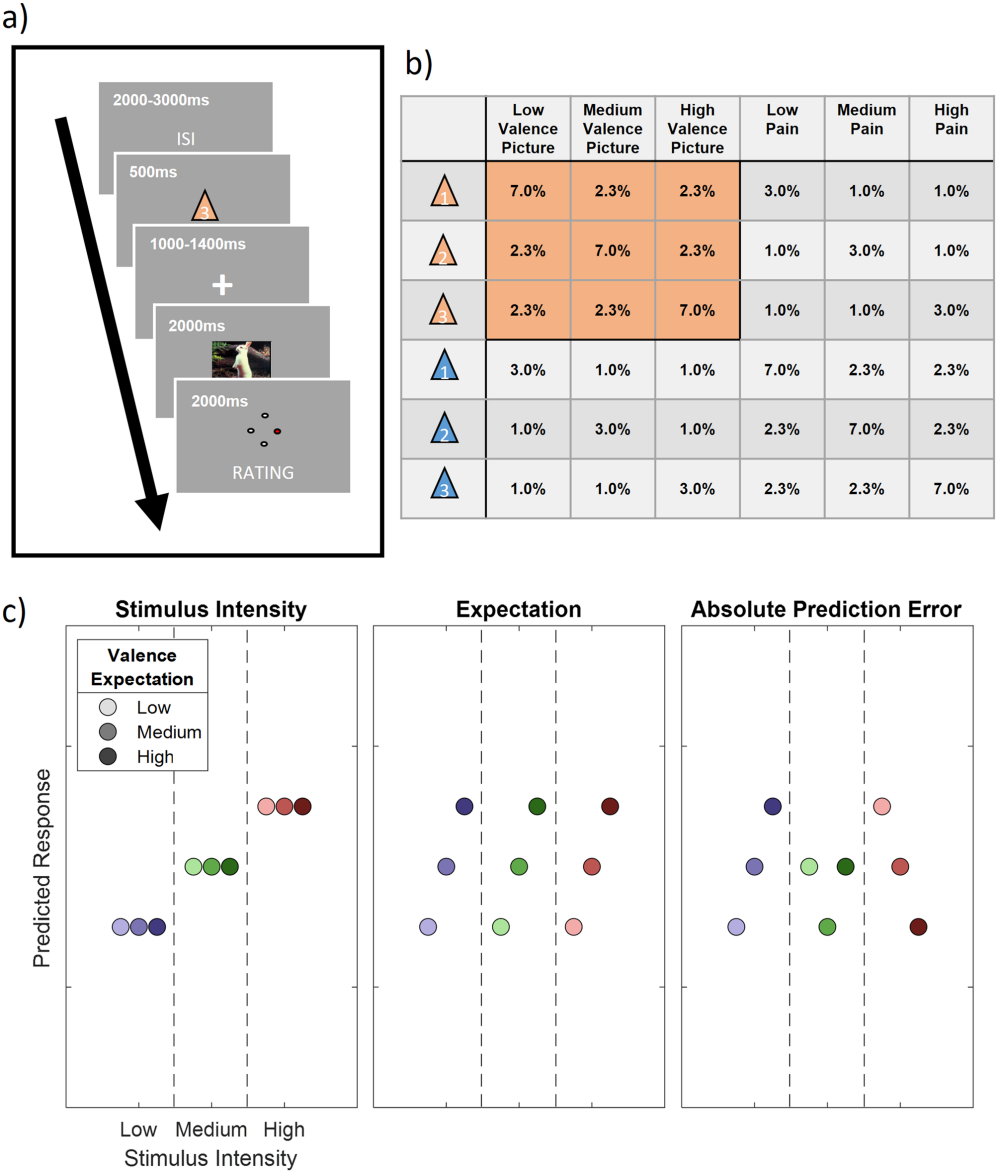
Overview of the study design. (**a**) Graphical representation of the trial structure. Each trial started with the presentation of a cue, indicating the stimulus intensity and modality of the following stimulus. After a jittered phase where only the fixation cross was shown, the stimulus (IAPS picture or pain) was presented. A rating phase (1–4) of the stimulus aversiveness followed. (**b**) Contingency table for all conditions for each cue-stimulus combination. Note that percentages are for all trials, therefore each row adds up to 1/6 (6 different cues). Orange fields indicate conditions included in the analysis, i.e. IAPS pictures where IAPS pictures were indicated by the color of the preceding cue. (**c**) Hypothetical response patterns based on Stimulus Intensity (INT; left), Expectation (EXP; middle) and Absolute Prediction Error (PE; right). The y-axis represents a hypothetical response variable (e.g. EEG power or rating). Each dot represents a different condition for each stimulus-cue combination. Blue colors represent low valence conditions, green colors represent medium valence conditions and red colors represent high valence conditions. Color intensities depict expectation level.

Each trial began with the presentation of the cue for 500 ms as an indicator for the modality and intensity of the subsequently presented stimulus. The modality (i.e. pain or picture) was correctly cued in 70% of all trials by the color of the triangle. In 60% of all trials, the stimulus intensity was correctly indicated by the digit within the triangle (see Fig. 1b for an overview of all cue contingencies).

Before the presentation of the stimulus, there was a blank period with a variable duration between 1000 and 1400 ms. The visual (or thermal) stimulus was presented for a duration of two seconds. The visual stimulus (horizontal visual angle of 3.8°; vertical visual angle of 2.4°) was centered on the screen and allowed the participant to perceive it without eye movements. After the termination of the stimulus, subjects were asked to rate the aversiveness of the stimulus on a four point rating scale, where 1 was labeled as “neutral” and 4 was labeled as “very strong”. Ratings were performed using a response box operated with the right hand (see Fig. 1a for a visualization of the trial structure).

In addition, four catch trials were included in each block. Subjects were asked to report the preceding cue in terms of their information content of the modality and intensity within 8 s and no stimulation was given in these trials.

Trials were presented in four blocks. Each block consisted of 126 trials and four catch trials and lasted about 15 min. The trial order within each block was pseudorandomized. The order of blocks was randomized across subjects. The whole EEG experiment including preparation and instructions lasted for about three hours.

Prior to the actual EEG experiment, subjects participated in a behavioral training session. During this session, they were informed about the procedure and gave their written informed consent. The behavioral training session was implemented to avoid learning effects associated with the contingencies between the cues and the stimuli during the EEG session. Between two and three blocks were presented during the training session (without electrophysiological recordings). The experimenter assessed the performance after each block based on the percentage of successful catch trials and the ability to distinguish the three levels of aversiveness of each modality. The training session was terminated after the second block if participants were able to successfully label cues in 75% of the catch trials within the second block.

### EEG data acquisition

EEG data were acquired using a 64-channel Ag/AgCl active electrode system (ActiCap64; BrainProducts) placed according to the extended 10–20 system^44^. Sixty electrodes were used of the most central scalp positions. The EEG was sampled at 500 Hz, referenced at FCz and grounded at Iz. For artifact removal, a horizontal, bipolar electrooculogram (EOG) was recorded using two of the remaining electrodes and placing them on the skin approximately 1 cm left from the left eye and right from the right eye at the height of the pupils. One vertical electrooculogram was recorded using one of the remaining electrodes centrally approximately 1 cm beneath the left eye lid and another electrode was fixated on the neck at the upper part of the left trapezius muscle to record an electromyogram (EMG).

### EEG preprocessing

The parameters and procedures for the EEG preprocessing were adopted from the analysis of the pain sub-data set for reasons of comparability and consistency (see Strube et al. 2021, https://elifesciences.org/articles/62809 to view detailed comments from reviewers on these pre-processing steps)^43^. The data analysis was performed using the Fieldtrip toolbox for EEG/MEG-analysis^45^. EEG data were epoched and time-locked to the onset of the IAPS picture. Each epoch was centered (subtraction of the temporal mean) and detrended and included a time range of 3410 ms before and 2505 ms after trigger onset.

The data were band-pass filtered at 1–100 Hz, Butterworth, 4th order. EEG epochs were then visually inspected and trials contaminated by artifacts due to gross movements or technical artifacts were removed. Subsequently, trials contaminated by eye-blinks and movements were corrected using independent component analysis (ICA) with a single ICA per subject for all trials concatenated^46,47^. In all datasets, individual eye movements, showing a large EOG channel contribution and a frontal scalp distribution, were clearly seen in the removed independent components. Additionally, time–frequency decomposed ICA data were inspected at a single trial level for micro saccades and muscle artifacts, after z-transformation (only for artifact detection purposes) based on the mean and the standard deviation across all components separately for each frequency from 31 to 100 Hz. Time–Frequency representations were calculated using a sliding window multi-taper analysis with a window of 200 ms length, which was shifted over the data with a step size of 20 ms with a spectral smoothing of 15 Hz. Gamma artifact components were easily visible and were compared with the trial-by-trial time series representations of all ICA components. Specifically, single and separate muscle spikes and micro saccades were identified as columns or “clouds” in time–frequency plots. Using this procedure, up to 31 components were removed before remaining non-artefactual components were back-projected and resulted in corrected data. Subsequently, the data was re-referenced to a common average of all EEG channels and the previous reference channel FCz was re-used as a data channel.

Before time–frequency transformations for data analysis were performed on the cleaned data set, the time axis of single trials were shifted to create separate cue-locked and stimulus-locked datasets. Cue-locked data defines the onset of the cue as t = 0. Stimulus-locked data defines t = 0 as the onset of the picture stimulus. Frequencies up to 30 Hz (1 to 30 Hz in 1 Hz steps) were analyzed using a sliding Hanning-window Fourier transformation with a window length of 300 ms and a step-size of 50 ms. It should be noted that delta and theta frequencies are not ideally mapped with these tapers because of a short window length. For the analysis of frequencies higher than 30 Hz (31 to 100 Hz in 1 Hz steps) spectral analyses of the EEG data were performed using a sliding window multi-taper analysis. A window of 200 ms length was shifted over the data with a step size of 50 ms with a spectral smoothing of 15 Hz. Spectral estimates were averaged for each subject over trials. Afterwards, a z-baseline correction was performed based on a 500 ms baseline before cue onset to avoid differences in the baseline based on modulations of the signal by the anticipation period. For cue-locked data, a time frame ranging from − 650 ms to − 150 ms was chosen as a baseline. A distance from the cue onset to the baseline period of 150 ms was set because of the half-taper window length of 150 ms, i.e. data points between − 150 ms and 0 ms are contaminated by the onset of the cue. For stimulus-locked trials, a variable cue duration (1500–1900 ms) was additionally taken into account, resulting in an according baseline from − 2550 ms to − 2050 ms from stimulus onset. For the baseline correction of time–frequency data, the mean and standard deviation were estimated for the baseline period (for each subject-channel-frequency combination, separately). The mean spectral estimate of the baseline was then subtracted from each data point, and the resulting baseline-centered values were divided by the baseline standard deviation^48^.

### Predictive coding model

Similar to a previous fMRI study^41^ and our analysis of the pain subset of this dataset^43^, our full model included three experimental within-subject factors (see Fig. 1c). The stimulus intensity factor (INT; see Fig. 1c; left column) models the measured response with a simple linear function of the stimulus intensity (− 1, 0 and 1 for low, medium and high intensities, respectively). The expectation (EXP) factor was defined (see Fig. 1c; center column) linearly from the intensity predicted by the cue. Again, conditions with a low intensity cue were coded with a − 1, conditions with a medium intensity cue with a 0 and conditions with a high intensity cue with a 1. The absolute prediction error factor (PE) resulted from the absolute difference of the expectation and actual stimulus intensity (see Fig. 1c; right column).

### Behavioral ratings

Behavioral aversiveness ratings were averaged for all 3 × 3 cue-stimulus combinations over each participant, resulting in a 29 × 9 matrix (subject × condition). We tested for main effects across stimulus intensity, expectation, as well as prediction error using one-way repeated measures ANOVAs as implemented in MATLAB (see fitrm and ranova, Matlab version 2020a, The MathWorks). Post-hoc tests were performed on the repeated measures ANOVA models using Bonferroni corrections for multiple comparisons as implemented in MATLAB (see multcompare, Matlab version 2020a, The MathWorks).

### EEG data analysis

The parameters and procedures for the EEG data analysis were adopted from the analysis of the pain sub-data set for reasons of comparability^43^. All statistical tests in electrode space were corrected for multiple comparisons using non-parametrical permutation tests of clusters^49^.

We explored positive and negative time–frequency patterns associated with our variations of stimulus intensity, expectation and absolute prediction errors using one-way repeated measures ANOVAs as implemented in the Fieldtrip toolbox. A statistical value corresponding to p = 0.05 (F(1,28) = 4.196) obtained from the repeated measures ANOVA for each factor was used for clustering. Samples (exceeding the threshold of F(1,28) = 4.196) were clustered in connected sets on the basis of temporal (i.e. adjacent time points), spatial (i.e. neighboring electrodes) and spectral (i.e.± 1 Hz) adjacency. Further, clustering was restricted in a way that only samples were included in a cluster which had at least one significant neighbor in electrode space, i.e. at least one neighboring channel also had to exceed the threshold for a sample to be included in the cluster. Neighbors were defined by a template provided by the Fieldtrip toolbox corresponding to the used EEG montage.

Cluster tests were applied separately for low frequencies (1–30 Hz in 1 Hz steps) and high frequencies (31–100 Hz in 1 Hz steps) in a time frame from 0 (onset of visual stimulus) to 2000 ms (end of visual stimulus presentation) for stimulus-locked data and from 0 (onset of cue) to 1500 ms (visual stimulus onset) for cue-locked data. Stimulus-locked data was tested for stimulus intensity, expectation and absolute prediction errors factors. Cue-locked data was tested for the expectation factor.

Subsequently, a cluster value was defined as the sum of all statistical values of included samples. Monte Carlo sampling was used to generate 1000 random permutations of the design matrix and statistical tests were repeated in time–frequency-channel space with the random design matrix. The probability of a cluster from the original design matrix (p-value) was calculated by the proportion of random design matrices producing a cluster with a cluster value exceeding the original cluster where a p-value < 0.05 indicated a significant difference. This test was applied two-sided for negative and positive clusters. Positive and negative clusters were determined by the fixed factor estimate (average slope of all subjects) resulting from a simple linear regression analysis of the respective main effect, i.e. an average decrease with factor levels was coded negatively whereas an increase was coded positively.

Clusters of activity reaching statistical significance (p < 0.05) were further evaluated using post hoc tests, which were applied on the mean value of all time–frequency-channel combinations included in the cluster using Bonferroni corrections for multiple comparisons as implemented in MATLAB (see multcompare, Matlab version 2020a, The MathWorks).

## Results

### Behavioral data

Participants experienced affective picture (or heat) stimuli which were probabilistically cued in terms of modality and intensity, evoking an expectation of modality and intensity. The subsequently applied stimuli were then rated on a visual analog scale (VAS) from 1–4. Our primary behavioral question was whether ratings are influenced by the experimental manipulation of stimulus intensity, expectation and absolute prediction errors.

To evaluate the main effects of stimulus intensity, expectation and absolute prediction error with regards to the valence of the IAPS pictures, we employed a repeated measures ANOVA of the behavioral data, which revealed significant effects for the main effect of stimulus intensity, i.e. the three levels of valence (F(1,28) = 762.10, p < 0.001). Post hoc analyses using the Bonferroni corrections for multiple comparisons for significance indicated that all three factor levels differed significantly, revealing higher ratings for high valence pictures (M = 2.98, SD = 0.40) vs medium valence pictures (M = 1.70, SD = 0.30) and medium valence pictures vs low valence pictures (M = 1.09, SD = 0.07; all p < 0.001).

**Figure 2:**
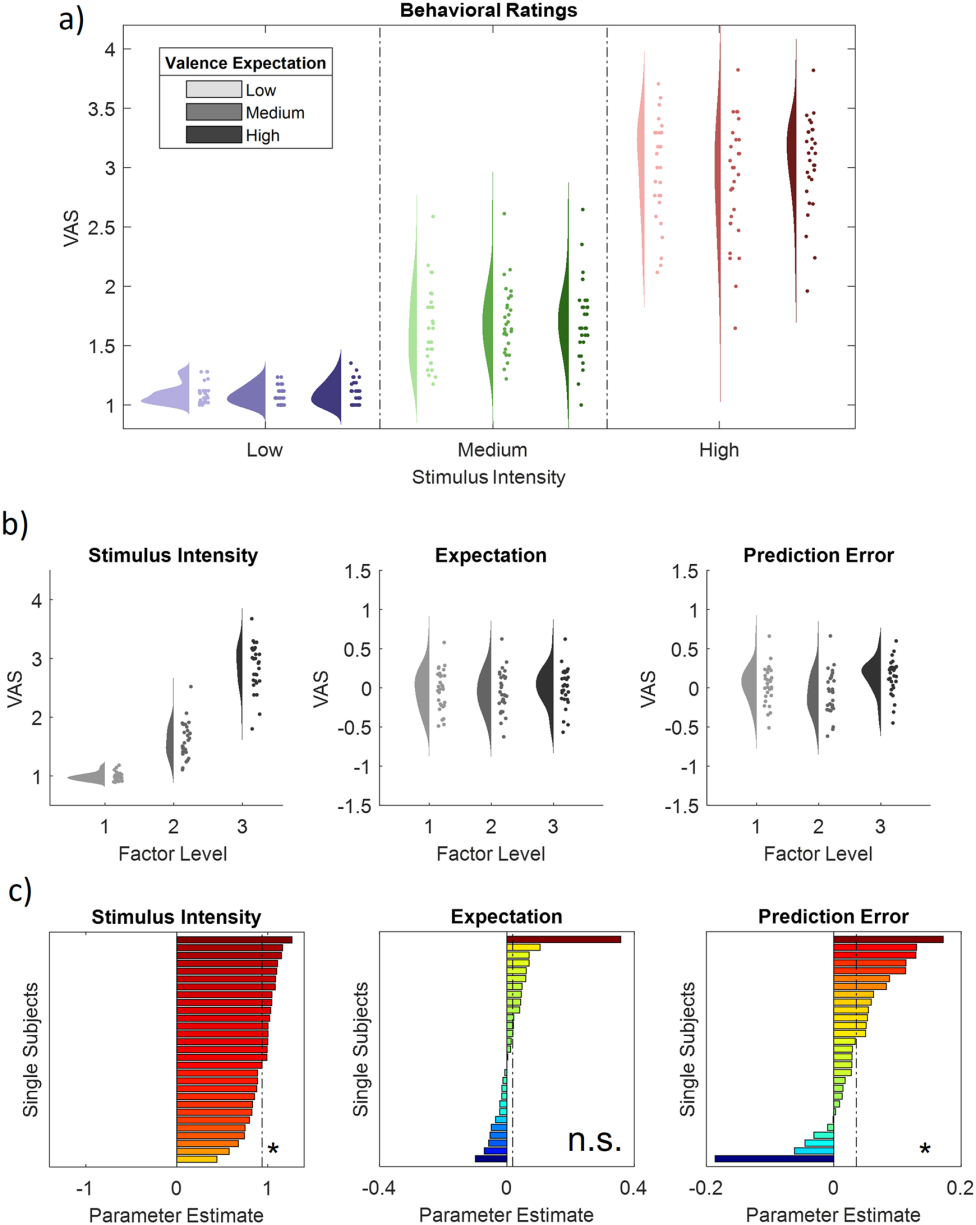
Ratings for IAPS picture stimuli. (**a**) Raincloud plots representing single subject ratings for all 9 congruent conditions (expect a picture and receive a picture). VAS (Visual Analog Scale) represents the rating on a 1–4 rating scale. Blue colors represent low valence IAPS picture stimuli, green colors medium valence IAPS picture stimuli and red colors high valence IAPS picture stimuli. The data show both an effect of stimulus intensity (increase from blue to green to red), but also a significant positive effect of absolute prediction errors. (**b**) Main effect plots for the stimulus intensity (F(1,28) = 762.10, p < .01), expectation (F1,28) = 1.46, p = .24) and prediction error (F1,28) = 7.7, p < .05) factors (from left to right) showing single subject values and distributions on the response, partialling out (for display purposes) the effects of the other predictors (e.g. EXP and PE were partialled out for the main effect plot of INT) for all three factor levels (increasing from left to right). (**c**) Bars represent the estimated slope for each subject and factor (stimulus intensity, expectation and prediction error from left to right). The dashed line represents the fixed factor estimate (average slope of all subjects). Hot colors represent a positive slope (increases with factor levels) and cold colors a negative slope (decreases with factor levels).

The main effect for expectation on aversiveness ratings did not yield a significant effect (F(1,28) = 1.46, p = 0.24). However, the absolute difference between the cued intensity and the actual stimulus intensity (i.e. absolute prediction error), showed a significant effect on aversiveness ratings (F(1,28) = 7.7, p = 0.01). Post hoc tests indicated that the condition without PEs (M = 1.95, SD = 0.24) was significantly smaller than the high PE conditions (M = 2.05, SD = 0.23; p < 0.001). Also, the low PE condition (M = 1.83; SD = 0.28) was significantly smaller than the no PE condition (p < 0.01). In summary, aversiveness ratings were increasing with the degree of aversive valence of the presented picture stimuli. Moreover, these results demonstrate higher ratings when there was a mismatch between the degree of aversion signalized by the preceding cue and the actual stimulus content, i.e. high prediction errors are related to higher aversiveness ratings. The results regarding picture stimuli are summarized in Table 1. See Fig. 2 for a descriptive rain cloud plot of behavioral ratings for each condition, main effects plot for each factor and single subject parameter estimates, showing significant positive intensity and prediction error factors.

**Table 1:** Main effects of stimulus intensity, expectation and absolute prediction errors on subjective aversiveness ratings in affective picture conditions.

Factor	Stimulus intensity (INT)		Cued intensity (EXP)		Absolute prediction error (PE)	
	*F(1,28)*	*p*	*F(1,28)*	*p*	*F(1,28)*	*p*
Behaviroal ratings	762.10	< .001	1.46	.24	7.7	.01

### EEG Intensity

EEG analysis were performed in the same way as the pain sub-data set^43^. We tested our EEG time–frequency data for a main effect of the valence of the aversive IAPS pictures in the context of a correctly cued modality (i.e. an IAPS picture was expected and received). In order to do so, we performed a repeated measures ANOVA on the time–frequency representation of the EEG data on low frequencies (1–30 Hz) and high frequencies (31–100 Hz) separately using a cluster correction criterion to address the multiple comparisons problem (see “Methods” for details). Any significant cluster—composed of neighboring data points in time, frequency and space—would indicate a neuronal oscillatory representation of variations in stimulus intensity in a given frequency band.

**Figure 3:**
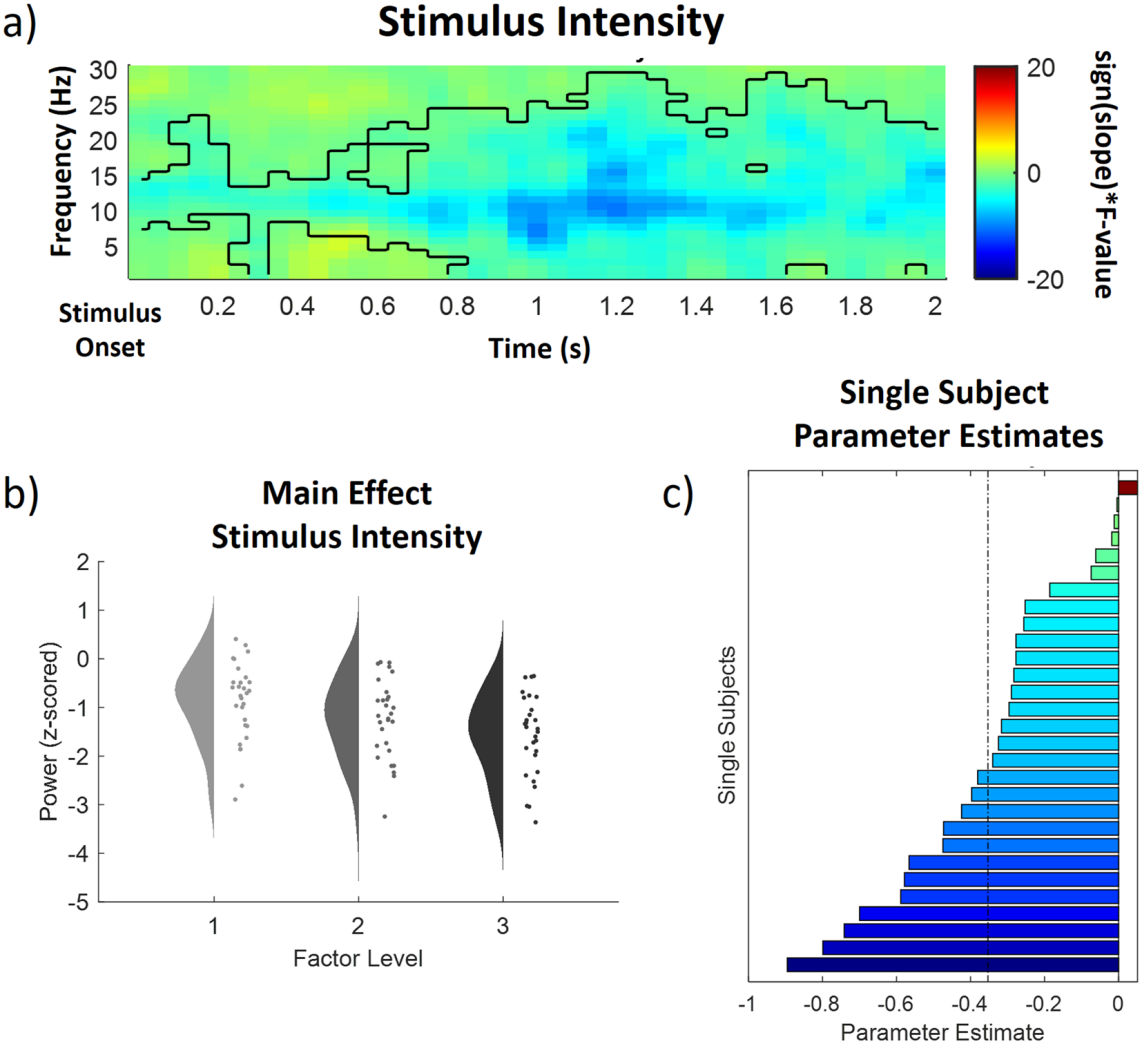
Time–frequency representation (**a**), main effect plot (**b**) and single subject parameter estimates (**c**) for the significant stimulus intensity (INT) cluster (p < .001, cluster-corrected), showing a decrease of alpha-to-beta power with an increased aversiveness of the stimulus. Time–frequency representations (**a**) are composed of the statistical F-values of the repeated measures ANOVA averaged over all channels. The significant cluster is outlined. Hot colors represent a positive slope (increases with factor levels) and cold colors a negative slope (decreases with factor levels). The main effect plot (**b**) for the INT cluster summarizes single subject values and distributions on the response, partialling out the effects of the respective other predictors (i.e. EXP and PE were averaged out for the main effect plot of INT) for all three factor levels (increasing from left to right). Single subject parameter estimates (**c**) are based on a linear regression of single subject values of each factor level. The dashed line represents the fixed factor estimate (average slope of all subjects).

In the low frequency (1–30 Hz) range, we observed one significant negative cluster of activity (p < 0.001) indicating a negative association of IAPS valence and power in the alpha-to-beta range (See Fig. 3 for a time–frequency representation, a main effects plot and single subject parameter estimates of the INT cluster). Specifically, this negative cluster included samples in a time range from 0 to 2000 ms after IAPS stimulus onset in a frequency range from 1 to 30 Hz. All channels included samples of the negative low frequency stimulus intensity cluster. Bonferroni corrected post hoc tests applied on the mean value of all time–frequency-channel combinations included in the INT cluster revealed that all comparisons, i.e. low valence (M = − 0.38, SD = 0.80) vs medium valence (M = − 0.85, SD = 0.80), medium valence vs high valence (M = − 1.08, SD = 0.82) and low valence vs high valence were significant (all p < 0.05), i.e. higher picture valence was related to lower alpha-to-beta power.

**Figure 4:**
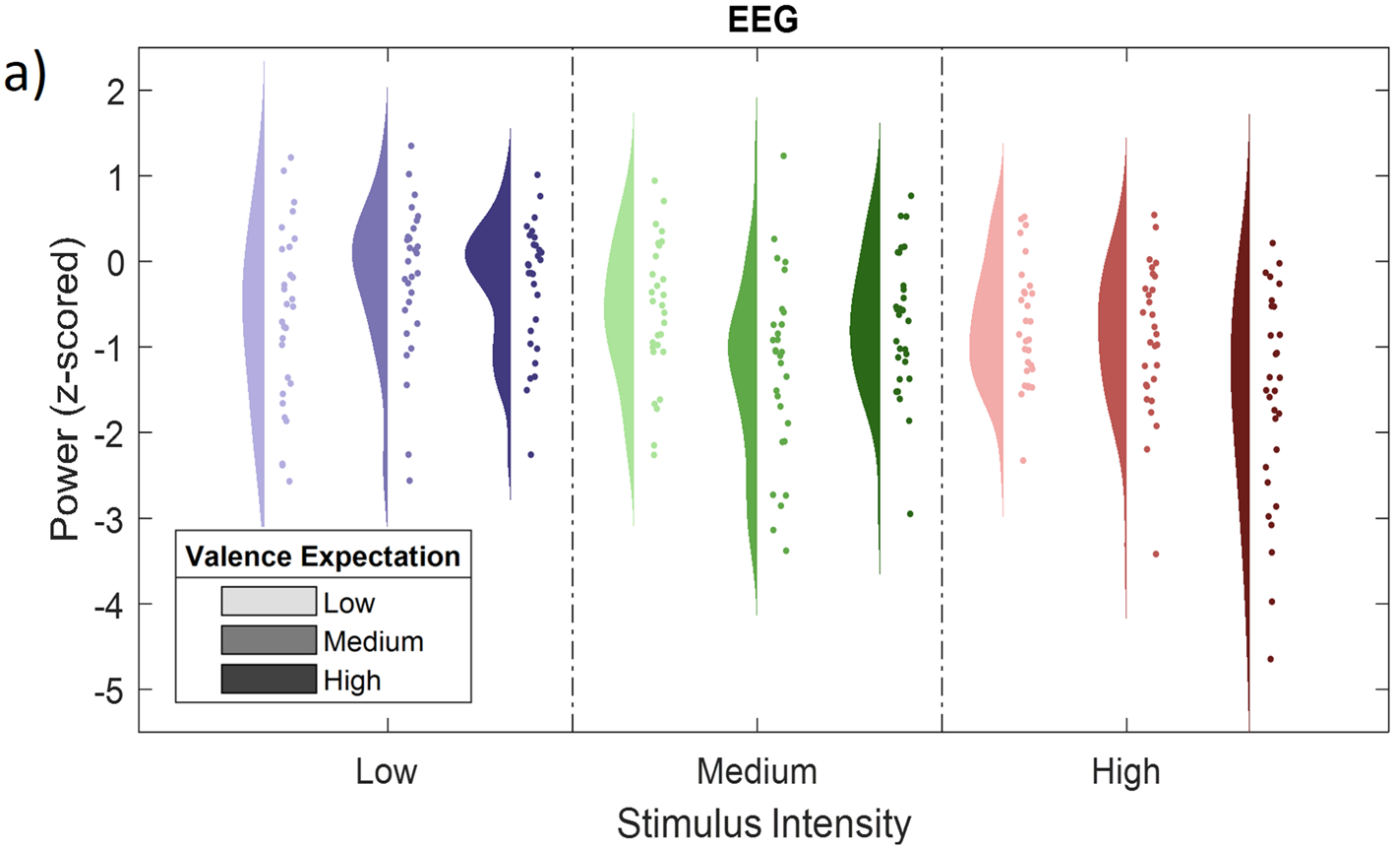
EEG activity at the significant INT cluster. (**a**) Scatter plots representing single subject EEG power (averaged over all samples included in the significant INT cluster) for all 9 congruent conditions (expect a picture and receive a picture) and according probability distributions averaged over all significant samples included in the negative INT cluster (0–2000 ms; 1-30 Hz).

In conclusion, these results indicate that a higher picture valence is associated with decreased alpha-to-beta band power (see Fig. 4 for a rain cloud plot of average EEG power at the INT cluster). No effect was observed for higher frequencies between 31 and 100 Hz.

### Expectation

In a next step, we investigated the representation of the expectation factor (EXP) in our repeated-measures model, again for low frequencies (1–30 Hz) and high frequencies (31–100 Hz) separately in the IAPS stimulus-locked and cue-locked time–frequency representation of the EEG data.

**Figure 5:**
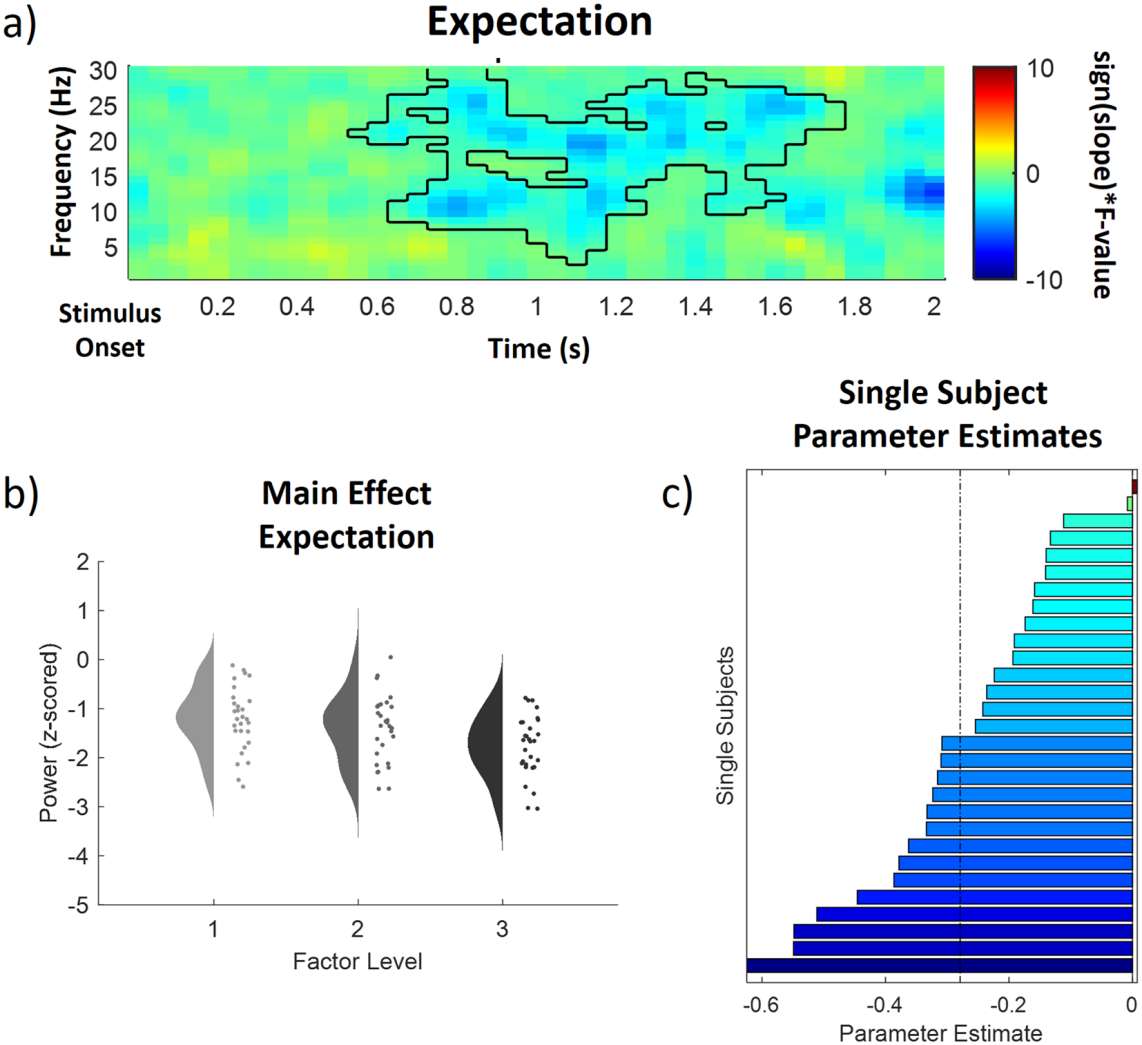
Time–frequency representation (**a**), main effect plot (**b**) and single subject parameter estimates (**c**) for the significant expectation (EXP) cluster (p < .05, cluster-corrected), showing a decrease of alpha-to-beta power with an increased expected valence of the stimulus. Time–frequency representations (**a**) are composed of the statistical F-values of the repeated measures ANOVA averaged over all channels. The significant cluster is outlined. Hot colors represent a positive slope (increases with factor levels) and cold colors a negative slope (decreases with factor levels). The main effect plot (**b**) for the EXP cluster summarizes single subject values and distributions on the response, partialling out the effects of the respective other predictors (i.e. INT and PE were averaged out for the main effect plot of EXP) for all three factor levels (increasing from left to right). Single subject parameter estimates (**c**) are based on a linear regression of single subject values of each factor level. The dashed line represents the fixed factor estimate (average slope of all subjects).

This analysis revealed one significant negative cluster in the low frequency range (1–30 Hz) after IAPS stimulus onset, indicating a negative association of cued intensity (EXP) and power in this frequency range (p < 0.05). The expectation cluster (p = 0.017) included samples from time points ranging from 550 to 1750 ms after IAPS stimulus onset and included frequencies from 3 to 30 Hz. All channels included samples of the negative low frequency EXP cluster (See Fig. 5 for a time–frequency representation, a main effects plot and single subject parameter estimates of the EXP cluster). Post hoc tests revealed that all comparisons, i.e. low valence expectation (M = − 0.77, SD = 0.65) vs medium valence expectation (M = − 1.11, SD = 0.68), medium valence expectation vs high valence expectation (M = − 1.33, SD = 0.62) and low valence expectation vs high valence expectation were significant (all p < 0.001, Bonferroni-corrected), showing higher valence expectation was related to lower alpha-to-beta power.

A cluster analysis of the expectation factor in cue-locked EEG data (from 1 to 30 Hz for low frequencies and 31–100 Hz for gamma frequencies; from 0 to 1500 ms), did not reveal any significant cluster of activity associated with changes in EXP (all p > 0.05,). See Supplementary Fig. 1 for time–frequency representations for low, medium and high valence expectation conditions.

In conclusion, these results indicate that a higher valence expectation is associated with decreased alpha-to-beta band power during stimulus presentation.

### Absolute prediction errors

Finally, we investigated the representation of absolute prediction errors (PE) in our repeated-measures model for low frequencies (1–30 Hz) and high frequencies (31–100 Hz) separately in the IAPS stimulus-locked time–frequency representation of the EEG data. This analysis revealed two significant adjacent positive cluster after IAPS stimulus onset, indicating a positive modulation of EEG power by absolute prediction errors (PE) (p < 0.05).

**Figure 6:**
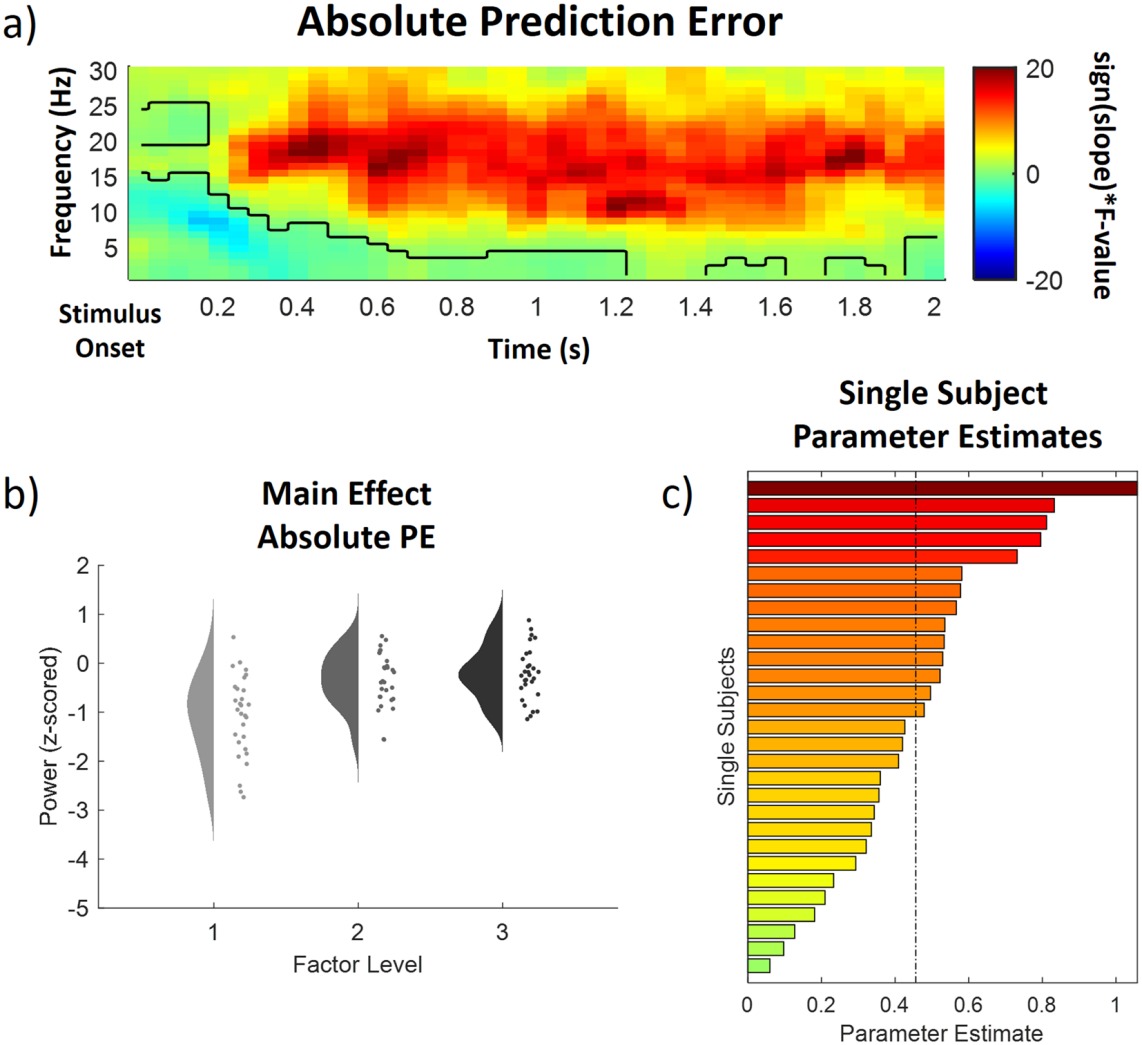
Time–frequency representation (**a**), main effect plot (**b**) and single subject parameter estimates (**c**) for the significant low frequency absolute prediction error (PE) cluster (p < .001, cluster-corrected), showing an increase of alpha-to-beta power with prediction errors. Time–frequency representations (**a**) are composed of the statistical F-values of the repeated measures ANOVA averaged over all channels. The significant cluster is outlined. Hot colors represent a positive slope (increases with factor levels) and cold colors a negative slope (decreases with factor levels). The main effect plot (**b**) for the PE cluster summarizes single subject values and distributions on the response, partialling out the effects of the respective other predictors (i.e. INT and EXP were averaged out for the main effect plot of PE) for all three factor levels (increasing from left to right). Single subject parameter estimates (**c**) are based on a linear regression of single subject values of each factor level. The dashed line represents the fixed factor estimate (average slope of all subjects).

One positive prediction error cluster was found in the low frequency range (1–30 Hz) (p < 0.001) and included samples from time points ranging from 0 to 2000 ms after IAPS stimulus onset and included frequencies from 1 to 30 Hz. All channels included samples of the low frequency absolute prediction error cluster (see Fig. 6 for a time–frequency representation, a main effects plot and single subject parameter estimates of the low frequency PE cluster). Here, post hoc tests revealed that conditions without prediction errors (M = − 1.55, SD = 0.82) were associated with significantly lower alpha-to-beta power than both, low (M = − 0.80, SD = 0.55) and high PE (M = − 0.68, SD = 0.54) conditions (all p < 0.001) whereas medium and high PE conditions did not differ in alpha-to-beta power (p = 0.1).

**Figure 7:**
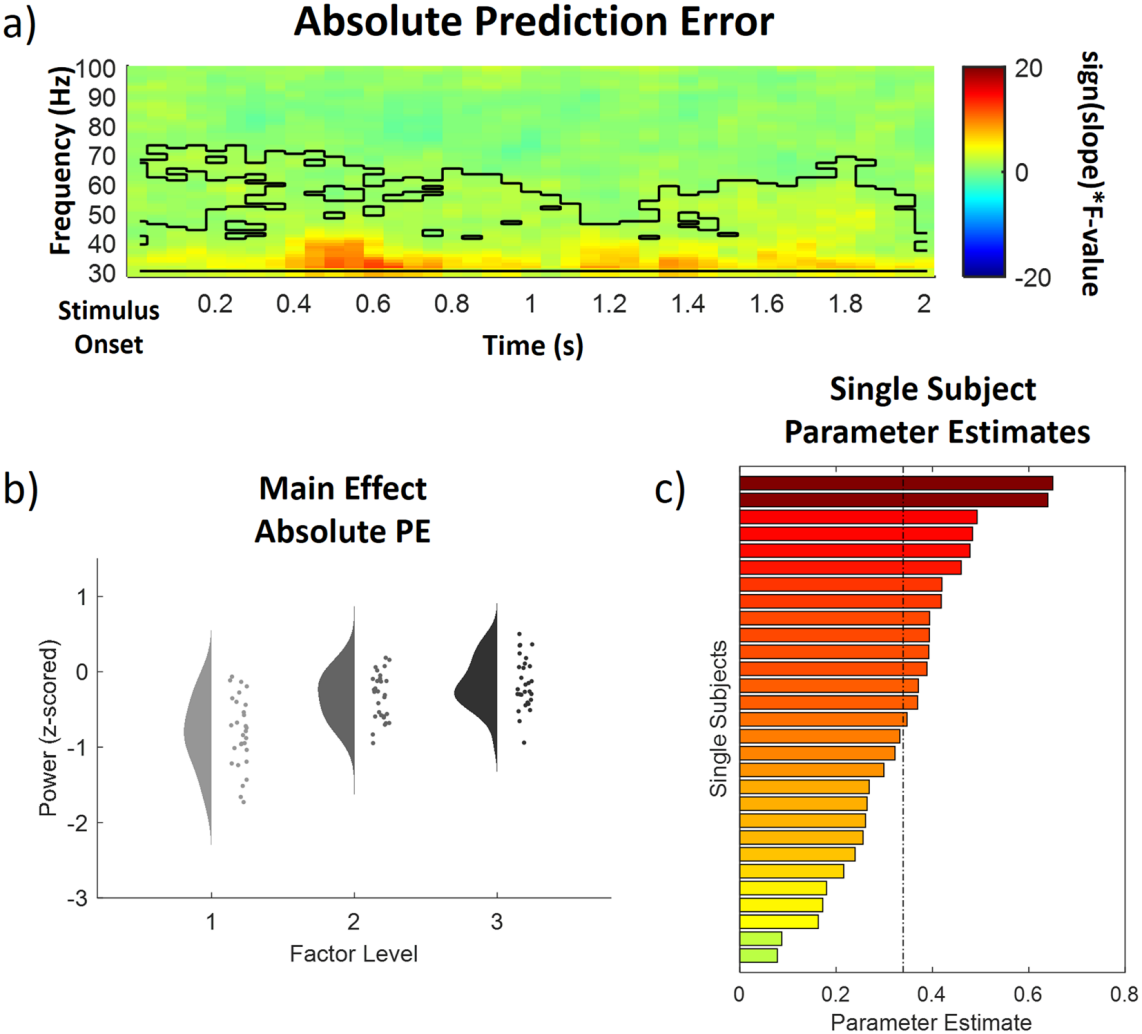
Time–frequency representation (**a**), main effect plot (**b**) and single subject parameter estimates (**c**) for the significant high frequency absolute prediction error (PE) cluster (p < .001, cluster-corrected), showing an increase of gamma power with prediction errors. Time–frequency representations (**a**) are composed of the statistical F-values of the repeated measures ANOVA averaged over all channels. The significant cluster is outlined. Hot colors represent a positive slope (increases with factor levels) and cold colors a negative slope (decreases with factor levels). The main effect plot (**b**) for the PE cluster summarizes single subject values and distributions on the response, partialling out the effects of the respective other predictors (i.e. INT and EXP were averaged out for the main effect plot of PE) for all three factor levels (increasing from left to right). Single subject parameter estimates (**c**) are based on a linear regression of single subject values of each factor level. The dashed line represents the fixed factor estimate (average slope of all subjects).

In the high frequency range (31–100 Hz) representing gamma activity one positive prediction error cluster was observed (p < 0.001) and included samples ranging from 0 to 2000 ms after IAPS stimulus onset and from 31 to 73 Hz. All channels included samples of the high frequency absolute prediction error cluster (See Fig. 7 for a time–frequency representation, a main effects plot and single subject parameter estimates of the INT cluster). Post hoc tests revealed that all comparisons were significant (all p < 0.01, Bonferroni-corrected) and conditions without PEs (M = − 0.84, SD = 0.46) were associated with a significantly lower gamma power than low PE conditions (M = − 0.36, SD = 0.31), and low PE conditions were associated with a lower gamma power than high PE conditions (M = − 0.19, SD = 0.34).

In summary, these results suggest an increase in alpha-to-beta and low gamma band power to be associated with expectation violations (i.e. absolute prediction errors), resulting from a mismatch of the cued intensity and the actual valence of the IAPS stimulus. Even though the parameters of our cluster analysis resulted in two separate clusters of activity, these clusters are connected in time, frequency and space which suggests this activity to be related to one single cluster.

## Discussion

Using a probabilistic cue paradigm with affective pictures of different valence levels, our data showed a clear discriminability of valence based on behavioral ratings and EEG time frequency patterns. Valence ratings were positively modulated by high prediction errors, supporting the hypothesis that prediction errors are linked to higher (negative) valence^40^. With regards to the EEG data, we observed one cluster of activity to be negatively correlated with the valence of the IAPS material in the alpha-to-beta band. Most importantly, our analysis also revealed expectations and violations of expectations (i.e. prediction errors) to be involved in the alpha-to-beta ERD and gamma power modulations.

Firstly, we hypothesized that alpha-to-beta ERD responses should be modulated by expectations. Additionally, we expected a modulation of these frequencies during the anticipation period from cue onset to the onset of the IAPS stimulus. Here, we found higher alpha-to-beta ERD associated with higher valence expectations during stimulus presentation, whereas we found no differences during the anticipation period.

Secondly, we hypothesized that surprise should lead to an increase of gamma ERS when there was a mismatch between the anticipated degree of aversion and the actual aversive quality of the picture. In contrast, if the negative valence or aversive quality is contributing to the gamma ERS effect, we expected an increase of gamma ERS with higher aversion regardless of the anticipated degree of aversion. Here, we provide evidence for gamma activity related to surprise as higher gamma power was associated with absolute prediction errors, whereas higher picture valence did not manifest in gamma power increases.

Our findings are in agreement with reports of decreases of alpha band power with unpleasant images and emotional arousal^19–28^. Even though many studies observed a decrease in power in the alpha- and lower beta band, some studies observed an increase with increased valence^28,30,50–52^. Interestingly, we found alpha-to-beta increases in power to be related to expectation violations as well as alpha-to-beta decreases associated with expected valence during the presentation of the IAPS stimulus. Anticipation of negative pictures enhances neural responses to the pictures^53,54^ during encoding of the emotional content, which is well in line with our findings of increased ERD with higher valence expectations.

Conversely, anticipation of aversive images did not manifest as differences after the presentation of the cue. It has been shown that in the anticipation period for affective images, alpha ERD preceding an anticipated negative image was larger as compared to a positive image^34^. Also, negative anticipation of affective images have been associated with the activation of the right prefrontal cortex in fMRI studies^55,56^. Interestingly, activation of brain areas associated with negative anticipation is decreased when anticipation of negative emotion is uncertain^57^. Here, all cues were to a large degree uncertain (after all, only 60% of all cues predicted the intensity correctly), which could explain that we could not detect expectation signals based on uncertainty of the anticipation. Alpha-to-beta band activity has been specifically implicated in the processing of top-down expectation signals^17,18^. Beta activity has also been linked to top-down prediction signals in the visual perception of causal events^58^. Here, we find alpha-to-beta activity associated with expectation signal only during stimulus presentation, suggesting that a representation of the prediction is reinstantiated during stimulus presentation.

EEG desynchronization is considered a reliable correlate of excited neural structures or activated cortical areas, while synchronization within the alpha band is hypothesized to be an electrophysiological correlate of deactivated cortical areas^59^ (see Pfurtscheller et al., 1996 for a review). An alternative view suggests increased alpha activity to be associated with active inhibition rather than passive inactivity^60–65^. More specifically, it has been suggested that alpha activity represents an attentional suppression mechanism when objects or features need to be specifically ignored or selected against^60^. Moreover, event related alpha synchronization is obtained over sites that probably exert top-down control and hence it has been assumed that alpha synchronization reflects a top-down process of inhibitory control^63^.

In this sense, inhibition is a mechanism for gating the flow of information throughout the brain which is mediated by alpha activity^61,62,65^. In our study, two effects come to play in the alpha-to-beta band, which are relevant with regard to this hypothesis: Firstly, alpha band activity shows a negative relationship with expected stimulus intensity, suggesting less inhibition (i.e. more attention to this information) of highly aversive (potentially negative or threatening) visual stimulation. Secondly, prediction errors resulted in increased alpha band power, i.e. a positive relationship. In this sense, incongruent trials would be attentionally suppressed and the features would be specifically ignored or selected against. This is because our alpha-to-beta prediction error follows a pattern of higher alpha-to-beta power with higher prediction errors. In this paradigm, the probabilistic characteristics of the cue did not change during the experiment and were learned before EEG measurements. It has been shown that the update of predictions is associated with beta ERD^66^. Here, an update of predictions based on unlikely events would reflect a change in predictions, even though the actual probabilities did not change. Following this thought, the update of predictions might be suppressed which manifests as higher alpha-to-beta power in prediction error conditions. This is in line with a proposed role of beta activity in actively maintaining the current cognitive set or the status quo^67^.

Beta oscillations have also been suggested to be associated with temporal reactivation of neural representations^68^. Beta modulations have been shown in working memory tasks, in which past information is brought into the focus of attention^68–70^. Here, conditions with prediction errors might be related to a similar process, where a mismatch was evaluated by a focus on the information of the cue stored in working memory. Our manipulation of prediction errors was associated with an increase in alpha-to-beta power: this suggests top-down processes (working memory and suppression) instead of bottom-up processes to be at play at alpha-to-beta frequencies associated with our prediction error factor.

In predictive coding, gamma activity has been specifically associated with prediction error responses^17,18^ and has been associated with bottom-up prediction errors in the visual processing of causal events^58^. Here, we found two clusters associated with prediction errors, firstly in the alpha to beta range and secondly in the gamma range. The gamma cluster needs to be interpreted with caution, as it might be affected by spectral smearing from the alpha-to-beta cluster.

In predictive coding, an improved causal model by learning improves top-down predictions which consequently lead to a reduction of bottom-up prediction error signals^71^. If we interpret both PE clusters (in the alpha-to beta range and in the gamma range) as incorporating different processes which are encoded simultaneously at different frequencies, our gamma PE cluster might be a manifestation of bottom-up prediction error signals. In predictive coding, gamma activity depends on the match between expectations and bottom-up input^17^ and is in this sense an assessment of sensory predictions^72^. In this study, we could directly assess the difference between expectations and bottom-up sensory input, resulting in differences in the gamma range. In summary, this would imply that top-down working memory demands and the suppression of prediction updates were encoded in the alpha-to-beta range whereas bottom-up prediction error signals were simultaneously encoded in the gamma range.

In the formulation of predictive coding, an important function of emotional valence turns out to regulate the learning rate of the causes of sensory inputs. Specifically it has been proposed that a violation of expectation leads to a (qualitatively) negative valence and an increase of the learning rate, while fulfilled expectations are associated with positive valence and a decrease of the learning rate^40^. Absolute prediction errors are also integral part of formal learning models. In the Pearce Hall model^73^, the absolute error promotes changes in associative strength (i.e. learning rate) such that large absolute prediction errors (surprises) prompt the model to rapidly adapt by increasing its learning rate. If emotions can be derived from a predictive coding function, visual processing of affective pictures can be seen as a simplified model of predictive coding processes in emotion.

## Limitations

This study has been designed in close analogy to a previous fMRI study to unravel the temporal dynamics of expectation and prediction errors and we decided to use the same experimental paradigm^41^. We therefore decided to also keep the sample characteristics similar and restricted the sample to male participants, which means that we cannot generalize our results to the population. Future studies should investigate samples including female participants. This would also allow to investigate sex effects with respect to expectation and prediction error effects in affective picture processing. The restriction to negative valence stimuli in this study limit the generalizability of our findings. Future studies could explicitly investigate positively valenced stimuli in the context of predictive coding.

## Summary

Our data show that key variables required for affective picture processing in the context of a generative model (i.e. predictive coding) are correlated with event-related alpha-to-beta and gamma activity. Alpha-to-beta activity was (negatively) modulated by valence expectations and stimulus valence, whereas prediction errors (positively) modulated responses from alpha-to-gamma frequencies. Alpha-to-beta increases associated with the mismatch of stimulus valence and expected valence imply working memory demands as well as the suppression of prediction updates, whereas gamma increases suggest a role of bottom-up processing of prediction errors.

## Supplementary Information

Supplementary Information.
