# Supplementary material for: Alpha-to-beta- and gamma-band activity reflect predictive coding in affective visual processing
Source: Sci Rep. 2021 Dec 6;11:23492. doi: 10.1038/s41598-021-02939-z (PMC8648824; doi:10.1038/s41598-021-02939-z)
Supplement: Supplementary file 1 — Supplementary Information. [file 41598_2021_2939_MOESM1_ESM.docx]

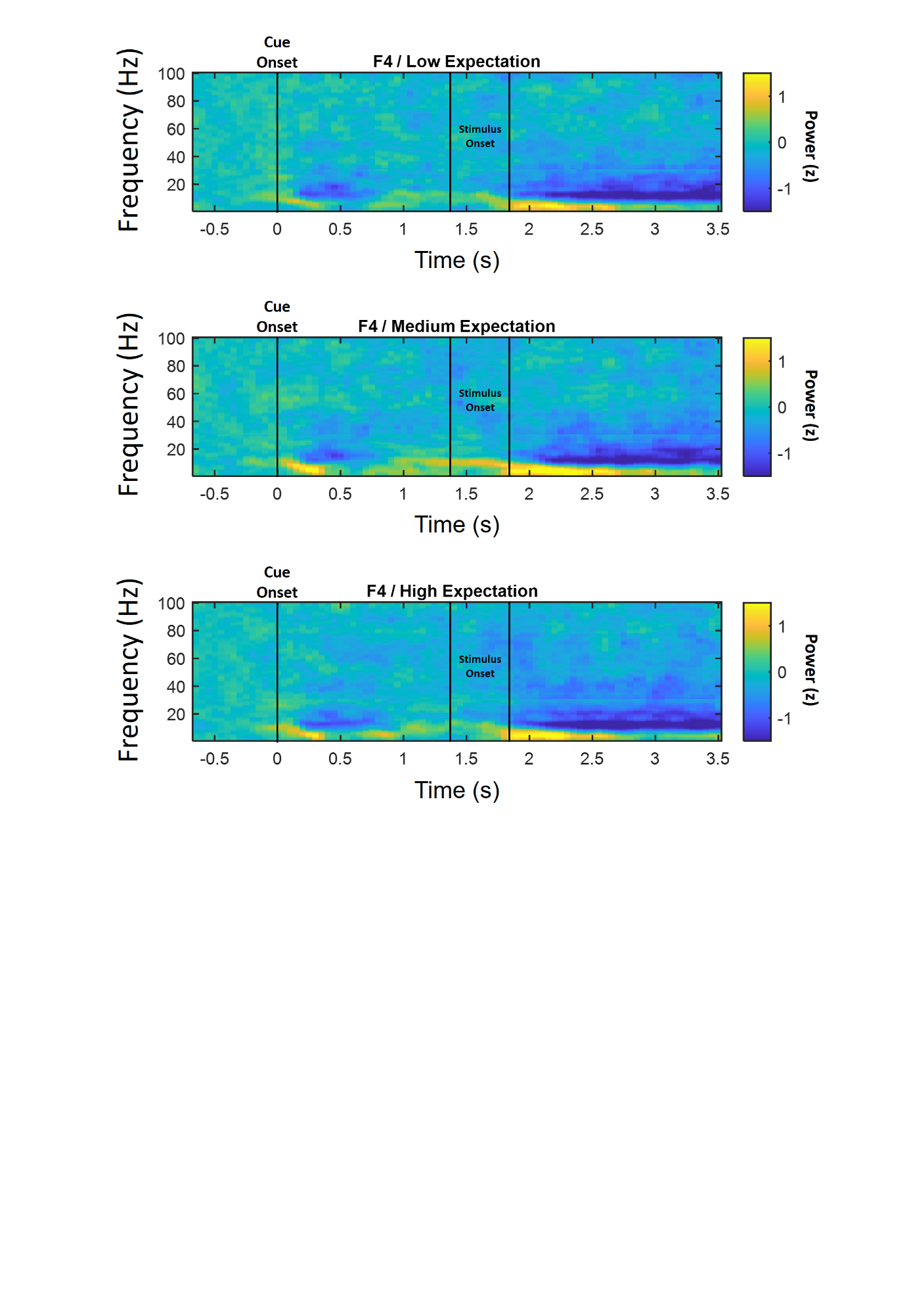


**Supplementary Figure 1.** Time-frequency representation of cue-locked data at F4 for low, medium and high valence expectation conditions. This figure was produced using Matlab version 2020a, The MathWorks.
